# CD4 regulatory T cells Control CD8 T cell responses to human Herpesvirus 8 lytic and latency proteins

**DOI:** 10.1186/1750-9378-7-S1-O17

**Published:** 2012-04-19

**Authors:** Lauren Lepone, Giovanna Rappocciolo, Paolo Piazza, Mariel Jais, Frank J Jenkins, Charles R Rinaldo

**Affiliations:** 1 Department of Infectious Diseases and Microbiology, University of Pittsburgh Graduate School of Public Health, Pittsburgh, PA, USA

## Objectives

CD8 T cells are considered to play an important role in controlling human herpesvirus 8 (HHV-8/KSHV) infection. However, these T cell responses are non-robust compared to other herpesviruses, suggesting that they are under tight regulatory control.

## Methods

Longitudinal PBMC samples were obtained from subjects with various outcomes of HHV-8 infection over many years in the Multicenter AIDS Cohort Study. The PBMC were tested by HLA A*0201 multimer staining specific for memory CD8 T cell epitopes of viral lytic and latency proteins, and polyfunctional flow cytometry to detect HHV8-specific, polyfunctional CD8 T cell populations. The effect of Treg was examined by depleting CD4^+^CD25^Hi^ cells.

## Results

Direct staining of PBMC with multimer MHC I complexes showed a relatively high frequency of circulating, HHV-8 lytic and latency antigen-specific CD8 T cells, but low anti-HHV-8 T cell polyfunctional reactivity. Removal of Treg enhanced T cell responses to these HHV-8 epitopes. The frequency of T cells specific for HHV-8 lytic antigens was greater than for latent antigens, and this effect was greater when Treg were removed. Numbers of HHV-8 specific effector memory CD8 T cells increased and terminally differentiated memory CD8 T cells decreased over many years of HHV-8 infection. We are currently assessing anti-HHV-8 T cell and Treg activity in relation to development of KS.

## Conclusions

We show for the first time that Treg suppress CD8 T cell responses to HHV-8 lytic and latency antigens, effectively masking more robust, underlying anti-HHV-8 T cell responses. The involvement of CD8 T cells and Treg in control of HHV-8 infection has important implications for development of vaccines to prevent KS.

